# Different time course of compensation of subjective visual vertical and ocular torsion after acute unilateral vestibular lesion

**DOI:** 10.1007/s00405-020-06312-0

**Published:** 2020-09-02

**Authors:** Mario Faralli, Giampietro Ricci, Leonardo Manzari, Giulia Zambonini, Ruggero Lapenna, Vito Enrico Pettorossi

**Affiliations:** 1 Department of Surgical and Biomedical Sciences, Section of Otorhinolaryngology, University of Perugia, Perugia, Italy; 2 MSA ENT Academy Center, Cassino, Italy; 3 Department of Experimental Medicine, Section of Physiology and Biochemistry, University of Perugia, Via Gambuli 1, Perugia, Italy

**Keywords:** Subjective visual vertical, Ocular cyclotorsion, Vestibular neuritis, Vestibular compensation, Otolithic receptors, Ocular tilt reaction

## Abstract

**Purpose:**

Time course of the recovery of otolithic dis-function caused by superior vestibular neuritis has been examined in fifteen patients.

**Methods:**

The subjective visual vertical (SVV) and the ocular cyclotorsion (OT) have been measured four times after the acute episode up to 1 year

**Results:**

In most of the patients the SVV tilt returned to control values within few months (3–6 months) after the acute episode, while OT remained out of normal range in almost all patients a year later.

**Conclusion:**

The abnormal OT observed after 1 year from the acute episode of vestibular neuritis, suggests that the otolithic receptors remained altered for several months and the OT may be a good indicator of the entity of the residual peripheral otolithic lesion. Moreover, the dissociation between the SVV tilt recovery and that of OT supports the issue that the two signs of the otolithic disfunction are only partially linked each other with centrally or peripherally distinct re-balancing circuits.

## Introduction

Ocular nystagmus, posture instability and vertigo are common symptoms after unilateral vestibular lesion [1–3]. Thereafter, the vestibular static and dynamic symptoms gradually attenuated together with the improvement of the quality of life [4, 5]. This process is due to peripheral recovery of the afferent activity or vestibular compensation, due to central adaptation taking place in the central nervous system. Vestibular clinical examinations among these patients have been traditionally based on the assessment of the signs of semicircular canal disfunction. However, the alteration of the otolithic receptors is also taken into consideration for fully evaluating the vestibular damage and the subsequent compensation [6]. The subjective visual vertical (SVV) [7, 8], the ocular tilt reaction [head tilt, skew deviation and ocular cyclotorsion (OT)] [9–12], vestibular evoked myogenic potentials (oVEMPs, cVEMPs) [12, 13] and the linear vestibulo-ocular reflex (LVOR) [14] are used for investigating the otolithic function, in combination with the test of the equilibrium using stabilometry tests. How otolithic functions recover overtime after unilateral vestibular damage or compensates the imbalance in the otolithic circuitry is still unclear. In particular, the evolution of the disfunction of the SVV and the cyclotorsion, two signs of static vestibular disfunction that are usually reported strictly related and in part dependent, is poorly investigated.

Normally, the subjective vertical line approaches the gravitational vector with good approximation and its deviation from the vertical is an important perceptual sign of otolithic tone imbalance in role plane [15, 16]. Ipsilesional deviation of the SVV indicates a damage within the peripheral vestibular system or lower brainstem [7, 9, 17, 18], contralateral deviation is due to lesion in a higher level in the central vestibular system. The correct orientation of SVV implies the high order cognitive process in which subjects use internal models to construct and update a sense of verticality based on multimodal sensory inputs of the vestibular system, proprioception and vision integrated by the central nervous system [16]. The OT toward the lesion side is also an important sign of otolithic tone imbalance in role plane. Both signs appear to be dependent on the imbalance of the basal activity between the two sides of the vestibular circuitry and influence each other since ocular torsion deviates the visual field alignment with gravity [7, 19–21].

In general, both the SVV and OT recover in conjunction after vestibular damage and faster compared with the semicircular canal functional deficit. It is reported that within 2–3 months the otolithic symptoms [22] fade away and at one year after the acute episode SVV and cyclotorsion returned to normal [23, 24].

However, it is reasonable to assume that signal processes for SVV and OT can evolve differently through different peripheral and central mechanisms. It is possible that SVV and OT may originate from distinct zones of the maculae. In a recent study, it has been shown discordance between ocular alteration of VEMPs and OT possibly due to different discrete zone of the utricle affected by the vestibular neuritis [25].

Moreover, the SVV appears a more complex neurological mechanism than the simple perceptual correlate of a vestibular-ocular reflex at the base of the OT. Vestibular cortical areas and the posterolateral thalamus seem to play a crucial role in this process of integration of vestibular and somatosensory input for the SVV, while the OT requires lower brain level of elaboration [18, 26–28].

Because of the possible separate peripheral and central pathways and different degree of multiple sensory integration we hypothesize that the progression of compensation after unilateral vestibular damage would show different time course. Dissociation of the recovery time course between reflex and perceptual vestibular responses has already been observed for the self-motion perception and VOR in the horizontal plane after vestibular neuritis. Indeed, the lesion induces an asymmetry of both horizontal VOR and motion perception, but VOR recovers earlier than the motion perception [29].

Since these reflex and perceptual otolithic clinical signs have not been accurately investigated during the process of compensation following unilateral vestibular damage, we planned a pivotal study for examining the attenuation of the otolithic signs after an episode of vestibular neuritis. We examined SVV and OT in the evolutionary stages in patients in which both signs were presently extending the period of observation up to one year.

## Materials and methods

Fifteen patients (9 male, 6 female) aged between 24 and 55 years (mean 42.80 ± 8.35) were enrolled in a prospective study. They came for the first time to our observation for the occurrence of acute dizzy symptoms during the period between 1 January 2016 and 31 December 2018. The otoneurologic investigation leads us to make a diagnosis of the unilateral vestibular lesion (UVL) [30]. In particular, the inclusion criteria were based on signs and symptoms of superior vestibular nerve neuritis (SVN) with acute onset of rotatory vertigo, nausea, horizontal spontaneous nystagmus (with a rotational component) toward the unaffected ear, postural imbalance with falls toward the affected ear, ipsilateral deficit in response to head impulse test and caloric irrigation, SVV tilt toward the lesion side and normal cVemps [30–32]. All these symptoms last several days. Patients with Meniere’s disease, benign paroxysmal positional vertigo, vestibular migraine and neurological disease emerged in the course of brain imaging (MRI, CT), were excluded from the study. Exclusion criteria included also: history of pre-existing abnormalities of eye movements (normal ductions and version, no history of strabismus), no history of ocular or orbital surgery, normal fundoscopic examinations, no high refractive defects, poor quality of fundus photographs. All patients have undergone to the same therapy which consisted of oral administration of corticosteroids (methylprednisolone) for 15 days according to the following scheme: 75 mg for the first 5 days, than 50 mg for a further 5 days, finally 25 mg for the other 5 days.

### SVV evaluation

We used 30 cm high fluorescent bar with LEDs, which was placed in a darkened room and set 1 m away from the subject [33, 34]. The bar (30 × 1 cm) was mounted on the wall via a central rode, around which the bar could be rotated by a remote control in both directions. At the upper end, there was a pointer that slided with the bar on a graduated scale, in which 0° corresponds to perfect alignment of the longitudinal line of the bar with the direction of gravity. Rotation of the pointer to the left corresponds to negative angles, whereas rotation to the right corresponds to positive angles. The entire bar was faintly illuminated. SVV was analysed with the subjects seated, in straight head position (0°). The bar was presented tilted 45° to the right or to the left and subjects should realign the bar according to its subjective vertical. Six measurements are conducted alternating the bar from a 45° angle to the right and then to the left, for a total of three measurements per side. The mean of six measurements with the subject’s head upright was the value taken into consideration.

### Ocular cyclotorsion *evaluation*

Ocular cyclotorsion (OT) was defined by analysing the ocular fundus performed in subjects with the head upright in the sitting position. Fundus Photographs were taken for both eyes separately during fixation of a central target (SPECTRALIS^®^, Heidelberg Engineering, Germany). OT indicates the position of the eye in the roll plane and was measured as the angle formed by a straight line passing through the papilla and fovea and a horizontal line. The angle value was expressed in degree. We defined ex-cyclotrope position the situation in which the horizontal plane passing through the fovea is located below the one passing through the papilla. Instead, in-cyclotrope position refers to the situation in which the horizontal plane passing through the fovea was above the one passing through the papilla. The final values of cyclotorsion of each eye is given by the mean of the angle obtained in six consecutive photshots to limit the effect of the torsional dynamic component of the spontaneous nystagmus on the static cyclotorsion. According to our previous study, to achieve a more precise measure of OT in the present study we take into account only the ocular torsion of the eye ipsilateral to the lesion. This measure has been considered less variable and more dependent on otolithic function [33, 34].

### Caloric test

Irrigation was performed with the patient supine and the head raised 30° according to Fitzgerald-Hallpike method, with water at 44 °C and subsequently at 30 °C for 40 s, 5 min apart. We measured peak slow phase eye velocity 60–90 s after irrigation onset and applied Jongkees formula for canal paresis [(right cold + right warm) – (left cold + left warm)] × 100/ (right warm + left cold + left warm + right cold)] and for directional preponderance [(right warm + left cold) – (right cold + left warm) × 100/(right warm + left cold + left warm + right cold)] [27, 35, 36].

#### Head shaking nystagmus test

With the patient seated and the head flexed 30°, the head was passively rotated horizontally by ± 45° at 1 Hz for 20 s, followed by EOG recording of any evoked nystagmus. The Head-Shaking Test was considered positive if the head shaking induced at least 2 clear post-rotation nystagmic beats with a peak slow phase eye velocity (SPEV) > 5°/s [37].

#### Control group

SVV and OT were also assessed in a control group of 15 healthy subjects matched for age and sex, not affected by vestibular diseases and ocular disorders. Among them, SVV and OT were found to be, respectively, 0.35 ± 0.96° and 6.42 ± 2.34° (ex-cyclotropic position).

#### Protocol

First the patients were evaluated with conventional vestibular tests for diagnosis of superior VN, otherwise known as Acute UVL. The evaluation took place within 48 h of symptoms onset. All patients underwent SVV and OT test at the first time of the diagnostic examination (1° control). The tests were repeated, respectively, three (2° control), 6 (3° control) and 12 months (4° control) after diagnosis.

### Statistics

The responses were statistically analysed by a Generalised Mixed Model Analysis (GLM) with SVV tilt angle or degree of ocular torsion as the dependent variables and group (patients and controls) and time of testing and interactions as the fixed effects of main interest and a random effect for the repeated measures. This analysis allowed to establish the statistical significance of the difference observed in patients and controls at different times of observation to find out whether and when patient values recovered to control values. Statistical post-hoc analysis was performed with Bonferroni’s post-hoc test for multiple comparisons. The level of significance was set at *p* < 0.05 for both the GLM values and post-hoc comparisons. Prior to GLM, the *W* test assessed normality and the Levene’s test of homogeneity of the variances. The regression line was also evaluated of the correlation between SVV inclination and the degree of ocular torsion at different times from the acute UVL episode.

The confidence interval (CI) of the values of vestibular tests was evaluated as 95% of the values resulting from our control group. All statistical evaluations and fittings were performed with the software OriginPro (Origin Lab Corporation, Northampton, MA, USA) and SPSS 16.0 IBM Corp. in Armonk, NY, USA.

## Results

### Subjective visual vertical

**Fig. 1:**
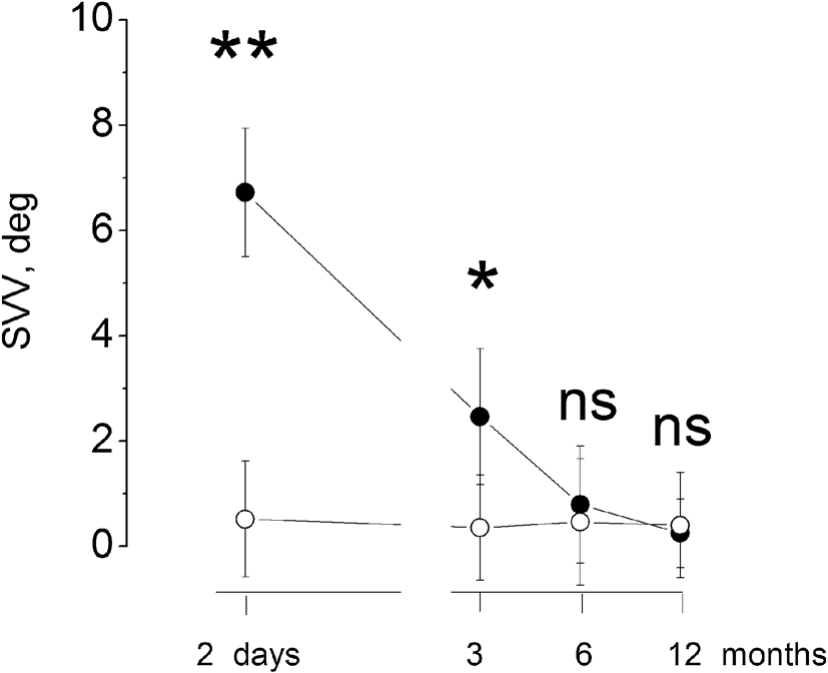
Tilt of the SVV after vestibular neuritis. Abscissa: time of observation after the acute episode, Ordinate: degree of tilt. The mean and SD are reported from 15 patients (filled circle) and 15 normal subjects (open circle). Double asterisk indicates the statistical significant *p* < 0.001, *0.01. *ns *not significant different

After the acute episode (1–3 days), the SVV tilt was 7 ± 1.23° toward the lesion side ranging from 4.95 to 8.95, significantly different from the inclination observed in our control group (0.12 ± 0.52°) (two way ANOVA: groups (patients/control): *F*_(1,28)_108, *p* < 0.001, partial *ƞ*^2^ = 0.84; time. *F*_(3,84)_ 171.1, *p* < 0.001 partial ƞ^2^ = 0.92, time × groups: *F*_(3,84)_ 170.4, *p* < 0.001, partial ƞ^2^ = 0.89) and all patients showed value above the upper confidence interval of our control group (values > 0.38, CI control). Thereafter, the SVV tilt decreased markedly within 3 months (2.85 ± 1.29, *p* < 0.01) reaching values lower than that of our control group at 6 months (0.79 ± 1.11, *p* = 0.32). and remained lower and 12 months (0.25 ± 0.65, patients vs control: *p* = 0.54). Only four patients showed values above the confidence interval of control at 6 months and two patients above the CI at 12 months (Figure 1).

### Ocular cyclotorsion

**Fig. 2:**
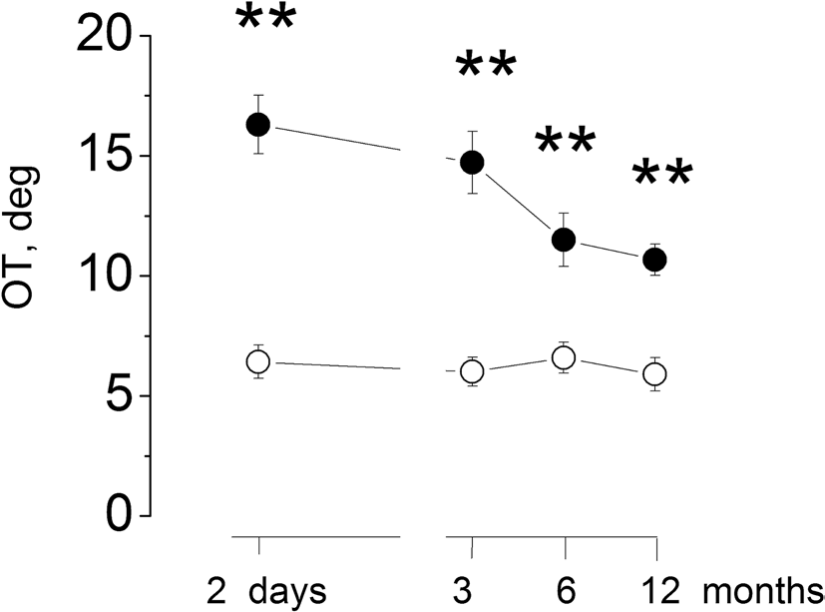
Ocular cyclotorsion after vestibular neuritis. Abscissa: time of observation after the acute episode, Ordinate: degree of the ocular torsion. The mean and SD are reported from 15 patients (filled circle) and 15 normal subjects (open circle). Double asterisk indicates the statistical significant *p* < 0.001

The ex-cyclotorsion toward the lesion side in the ipsilateral eye was of 16.33 ± 2.81° at the first examination 1–3 day after the acute episode, significantly higher than that of our control group (6.45 ± 0.92) (two way ANOVA: groups (patients/control): *F*_(1,28)_128.5, *p* < 0.001, partial ƞ^2^ = 0.87; time. *F*_(3,84)_ 38.9, *p* < 0.001 partial ƞ^2^ = 0.89, time × groups: *F*_(3,84)_ 20.9, *p* < 0.001, partial ƞ^2^ = 0.91). The amount of OT decreased gradually at 3 months (14.72 ± 2.7, *p* < 0.01and continue to decrease at 6 (11.51 ± 2.27, *p* < 0.01) and 12 months (10.68 ± 2.32). This torsion value remained significantly higher than the control value, *p* < 0.01. At the last measure only three patients showed values below the upper CI of the control (CI 7.21) (Fig. 2).

**Fig. 3:**
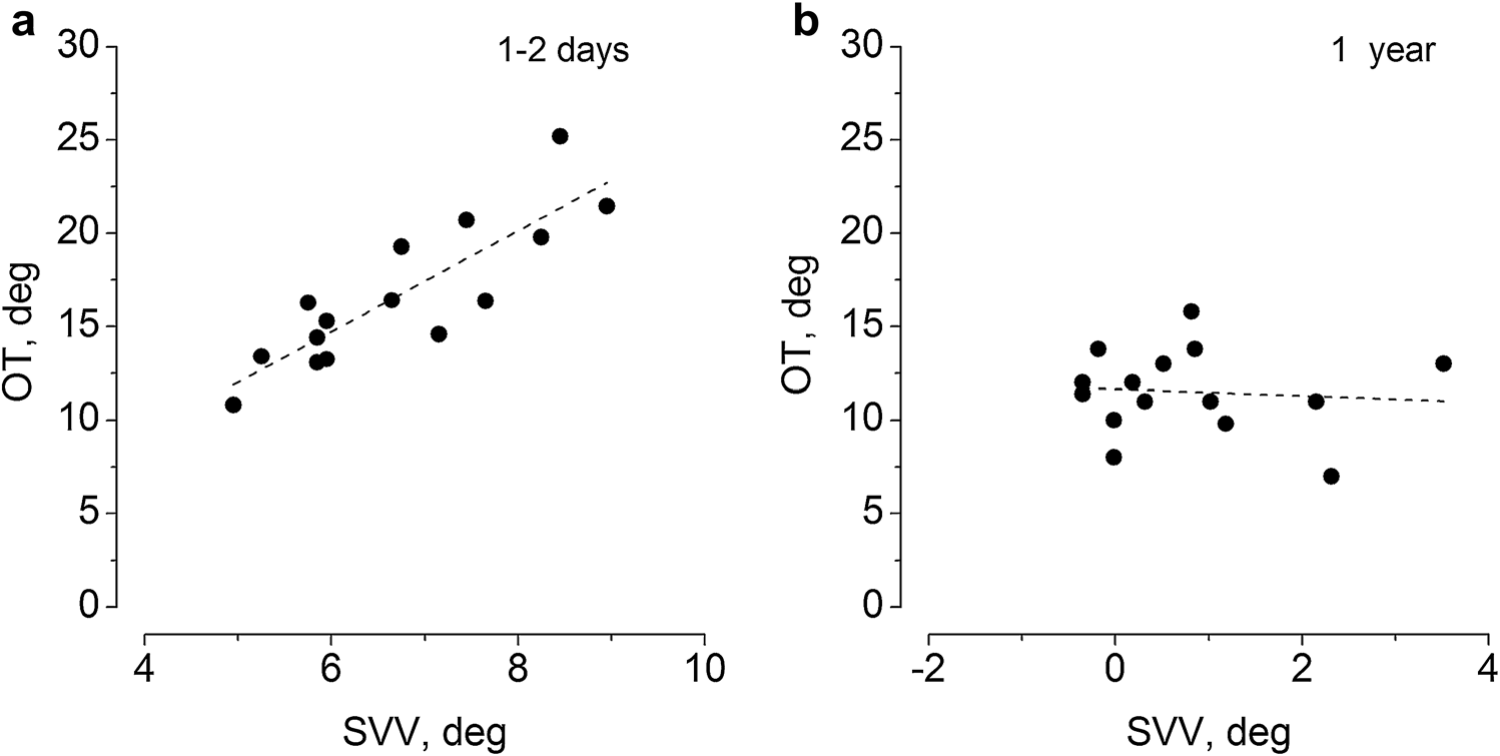
Correlation between the degree of ocular torsion and the SVV tilt immediately after the acute episode (1–2 days) (**a**) and 12 month after (**b**). Observation from 15 patients. In **a** there is a good correlation, while in **b** no correlation was observed. Dashed line represents the regression line: *Ad.* *R* square = *R*

Comparing the SVV and OT over the compensatory period we found correlation between SVV and OT at the first measure (*R* = 0.71, *p* < 0.01). After 12 months there was no correlation (*R* = − 0.07, *p* = 0.8–0.6) (Fig. 3).

### Canal paresis, directional preponderance and HST

**Fig. 4:**
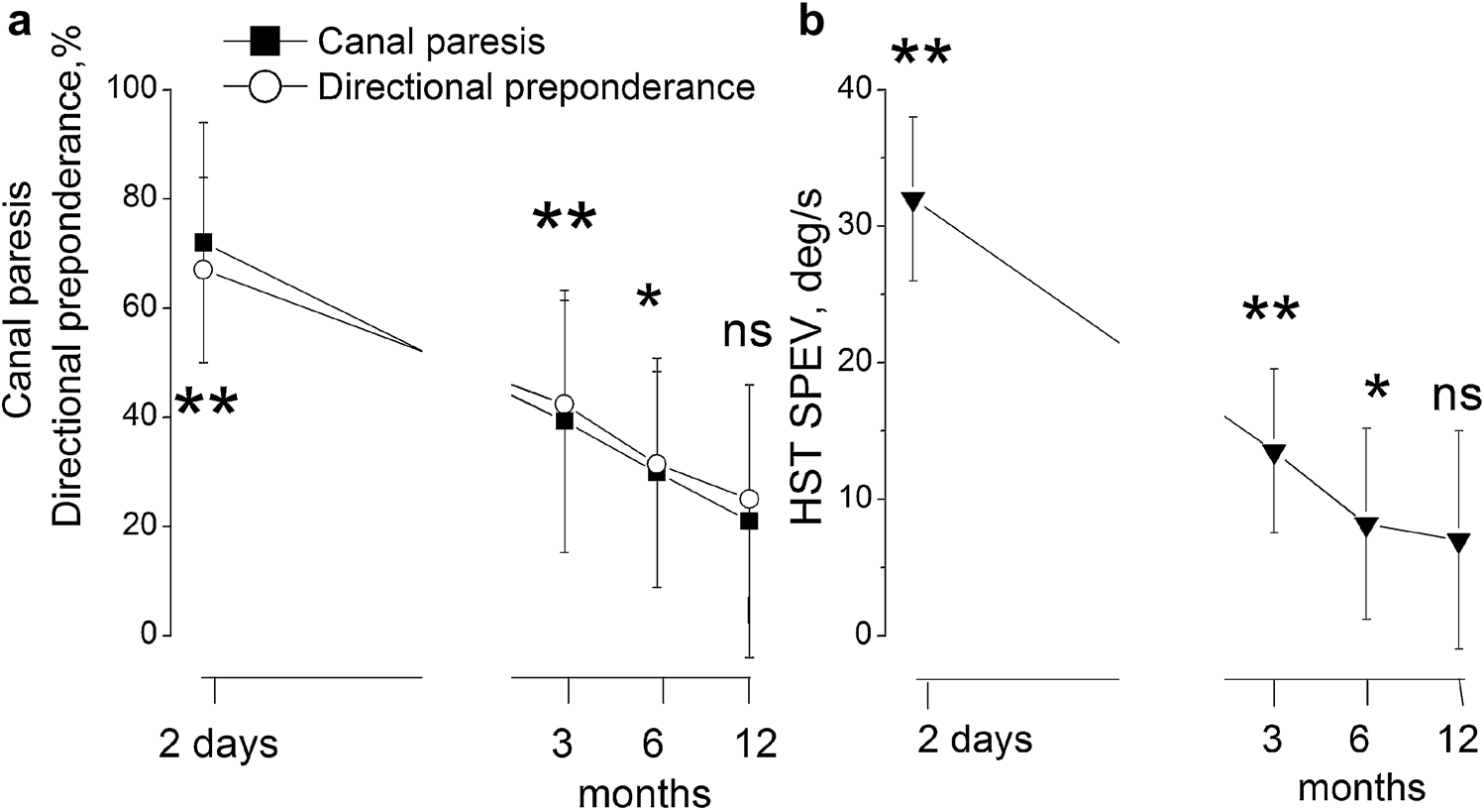
Canal paresis and directional preponderance in response to caloric stimulation after vestibular neuritis (**a**) and slow phase velocity of nystagmus induced by HST (**b**). Abscissa: time of observation after the acute episode, ordinate: **a**: % value of canal paresis (filled square) and of directional preponderance (open circle). B: slow phase velocity (deg/s) of the nystagmus induced by HST (filled triangle). The mean and SD are reported from 15 patients (filled circle) and 15 normal subjects (open circle). Double asterisk indicates the statistical significant *p* < 0.001, *0.01, *ns* not significant different

The responses to caloric test and HST were also examined in these patients. We calculated the mean and 95% CIs for canal paresis (CP) and the directional preponderance (DP)and the slow phase eye velocity of head-shaking nystagmus tests At the first measure after the acute episode the canal paresis (CP) and the directional preponderance (DP)  were markedly abnormal in all patients, evidencing a large asymmetry in the ocular responses to opposite side stimulation. All values were outside the CI of the control group. The nystagmus was elicited by the HST with high slow phase velocity (SPEV > 2–5°/s). Three month later, the asymmetry in response to caloric stimulation decreased and the occurrence and velocity of nystagmus by HST was also reduced. The values of CP and DP returned within the CI of control in 7 subjects at 6 months and in 12 subject at 12 months. Also the nystagmus velocity was reduced to null in the 10 patients. The GLM analysis for caloric, head shaking tests: caloric test: groups (patients vs controls): CP: group *F*_(1,28)_ = 72.5, *p* < 0.001,  partial *ƞ*^2^ = 0.86, testing time *F*_(3,84)_  = 15.1, *p* < 0.01, partial *ƞ*^2^ = 0.55 and interaction group × time *F*_(3,84)_ = 21.9, *p* < 0.001, partial *ƞ*^2^ = 0.86; DP: *F*_(1,28)_ = 38.5, *p* < 0.001,  partial *ƞ*^2^ = 0.73, testing time *F*_(3,84)_  = 9.6, *p* < 0.01, partial *ƞ*^2^ = 0.72 and interaction group × time *F*_(3,84)_ = 16.3, *p* < 0.001, partial *ƞ*^2^ = 0.78; Head shaking test: groups (patients vs controls) *F*_(1,28)_ = 64.7, *p* < 0.001, partial *ƞ*^2^ = 0.77, testing time *F*_(3,84)_ = 10.1, *p* = 0.01, partial *ƞ*^2^ = 0.72and interaction group × time *F*_(3,84)_ = 15.4, *p* < 0.001, partial *ƞ*^2^ = 0.69; post-hoc analysis showed that the asymmetry values were similar to the control value at 12 months for both the asymmetry of caloric stimulation and for the nystagmus velocity (*p* = 0.7–0.2) (Fig. 4).

## Discussion

This study investigated in VN patients the time course of the compensation of the SVV and OT. We found that both the amount of SVV tilt and of OT caused by unilateral vestibular damage gradually diminishes during the compensatory period but the time course of their attenuation is clearly dissociated. In fact, in most of the cases the SVV tilt values reached the range of normality in a few months, while the OT remained significantly abnormal at 1 year from the acute episode.

The timing of normalization of the SVV tilt is in agreement with other observations [22, 23], but the slower normalization of OT has not  been reported before. It is possible that the lack of this evidence is due not only to few studies devoted to this issue but also to the method used  not sufficiently accurate  for analyzing the ocular cyclotorsion. In fact, the accurate measure has been shown to be performed on the eye ipsilateral to the lesion since the value of the OT in the ipsilateral eye is much less variable compared with that observed in the contralateral eye [33, 38]. The greater variability of the torsion of the contralateral eye might indicate that the influence of the activated otolithic receptors is prevalently directed toward the ipsilateral eye.

The abnormal OT observed 1 year after the acute episode excludes the full recovery of the otolithic function suggesting that damage of specific area of the otolithic receptors involved in the OT is persistent. Conversely, the analysis of SVV suggests a complete functional recovery. This discordance might be attributed to the possibility either that distinct otolithic receptors contributing to the two different functions, are differently affected by the VN. The recent evidence of a discordance between the oVEMs alteration and the OT might suggest that different susceptibility of a different area of the otolithic receptors [25] and, therefore, of their recovery. Unfortunately, information on the oVEMPs at one year after the acute episode is not available. A clinical observation [39] suggests that the oVEMPs return to normal in some patients. However, we believe that a more extended examination is needed to ascertain the normality of the oVEMPs.

Another reason for the difference in the attenuation of the SVV and OT deficits may be due to distinct central areas and mechanisms guiding to functional recovery.

It is likely that two symptoms depend on the side imbalance of the otolithic peripheral signals, but the attenuation of the OT may result from a remodelling of the low brain stem circuits, while the attenuation of the SVV tilt may result from adaptive mechanisms involving more high and complex circuits. In the latter case, other sensory signals converge in this circuit so that vicarious extra-vestibular inputs can substitute the information in partial absence or alteration of the otolithic input. It is possible, in fact, that the proprioception may play a relevant role in reconstructing centrally a correct perception of the SVV. To support this point, it has been demonstrated that changes of the proprioception by high-frequency vibratory stimulation or of the support surface [40, 41], can modify SVV both in normal subjects and in unilateral vestibular neuritis patients when applied to neck muscles. Moreover, the erroneous perception of the vertical by side imbalanced of the otolithic signals will determine a conflict of visual and vestibular perceived vertical, so that the central nervous system (CNS) erroneously computes the vertical. The mismatch caused remarkable behavioural problems and these disturbing errors may leading the CNS to a quick and powerful re-organization for reassuming a correct perception of vertical. This quick correction is fundamental for re-adjusting posture, equilibrium and improving voluntary movements. Conversely, the functional error caused by the ocular torsion has a minor impact. In fact, visual acuity is practically uninfluenced by the ocular torsion, causing a very slight effect in term of diplopia. The slight visual error occurs even in normal subjects when tilting the head and, even in the normal this error is poorly perceived. This means that the error signal induced by incorrect eye torsion is much less distressing. Undeniably, the ocular torsion is in part responsible of the SVV [19] and of the consequent disfunction, but compensation of the SVV would annulate the effect of the OT into the SVV. Therefore, it is likely that a poor error signal induced by OT, compared to the error signal induced by SVV, would not drive powerfully the mechanism of compensation within the CNS.

Interestingly, since it is the skew deviation mostly responsible  of  diplopia [42, 43] we expect that this symptom would be more efficiently compensated than the OT. We have not carefully examined the skew deviation, but our clinical observation in peripheral vestibular lesion seems to indicate that the ocular dis-alignment is much less evident than the ocular torsion. This should be confirmed by a more accurate analysis.

Comparing the compensation of the OT and the ocular responses to conventional vestibular tests, the value of OT of the entire patient group remains significantly higher than the control 1 year after the acute episode, while the ocular responses to caloric stimulation and the HST nystagmus are not statistically different from the normal values, even though a large variability of values has been observed among the patients. The persistent OT alteration indicates that residual deficits remain in otolithic circuits, as a residual deficit persists in the response of the dynamic activation of the semicircular canals as shown by head trust test [23].

In conclusion, the analysis of the inclination of the SVV and the degree of OT during the post-lesion interval supports the issue that the two signals of the otolithic disfunction are only partially linked each other with centrally separate controlling circuits. This is even more evident after vestibular neuritis since the two circuits controlling SVV and OT appear to be individually attenuated with different time course. In addition, the OT, that seems to be scarcely compensated by the central process, may be a good indicator of the entity of the residual peripheral otolithic lesion.
